# Prognostic factors and predictors of outcome in patients with COVID-19 and related pneumonia: a retrospective cohort study

**DOI:** 10.1042/BSR20203455

**Published:** 2020-12-04

**Authors:** Gianluca E.M. Boari, Giulia Chiarini, Silvia Bonetti, Paolo Malerba, Gianluca Bianco, Cristina Faustini, Federico Braglia-Orlandini, Daniele Turini, Vittoria Guarinoni, Michele Saottini, Sara Viola, Giulia Ferrari-Toninelli, Giancarlo Pasini, Cristina Mascadri, Bianca Bonzi, Paolo Desenzani, Claudia Tusi, Eros Zanotti, Matteo Nardin, Damiano Rizzoni

**Affiliations:** 1 Division of Medicine, Covid-19 Unit M, Spedali Civili di Brescia, Montichiari, Brescia, Italy; 2 Department of Medical and Surgical Sciences, University of Brescia, Brescia, Italy

**Keywords:** ACE-inhibitors, cardiovascular risk factors, COVID-19, enoxaparin, hypertension, SARS-CoV2

## Abstract

The aim of the present study was to simultaneously assess several potential predictors of outcome (co-morbidity, previous and in-hospital treatment, radiologic Brixia score) in patients with COVID-19.

This retrospective cohort study included 258 consecutive patients with confirmed COVID-19 admitted to a medical ward at Montichiari Hospital, Brescia, Italy from February 28th to April 30rd, 2020. Patients had SARS-CoV-2 related pneumonia with respiratory failure, and were treated with hydroxychloroquine and lopinavir plus ritonavir. In some patients, additional treatment with tocilizumab, dexamethasone and enoxaparin was adopted. Outcomes (death or recovery) were assessed at the end of the discharge period or at the end of the follow-up (August 2020).

During hospitalization, 59 patients died, while 6 died after discharge. The following variables were demonstrated to be associated with a worse prognosis: Radiologic Brixia score higher than 8, presence at baseline of hypertension, diabetes, chronic obstructive pulmonary disease, heart disease, cancer, previous treatment with ACE-inhibitors or anti-platelet drugs. Anticoagulant treatment during hospital admission with enoxaparin at a dose higher than 4000 U once daily was associated with a better prognosis.

In conclusion, our study demonstrates that some co-morbidities and cardiovascular risk factors may affect prognosis. The radiologic Brixia score may be a useful tool to stratify the risk of death at baseline. Anticoagulant treatment with enoxaparin might be associated to a clinical benefit in terms of survival in patients with COVID-19.

## Introduction

Coronavirus disease 2019 (COVID-19) outbreak represented a major clinical problem in Lombardy, one of the most affected Italian (and worldwide) Regions by the epidemic. The pandemic emergency due to the rapid worldwide spread of SARS-CoV2 infection has promoted the need of an in-depth analysis of the predicting factors determining prognosis of patients that develop interstitial pneumonia with respiratory failure. It was suggested that hypertension, diabetes and other cardiovascular disorders may be substantially associated with SARS-CoV-2 infected patients and with poor outcome [1]. Furthermore, the severity of the primary respiratory syndrome is increased in patients with pre-existing cardiovascular disease [2]. SARS-CoV2 infects target cells by interacting with the converting enzyme of angiotensin 2 (ACE2) that is expressed in various organs including the lung, the heart, the kidney and the gut. ACE2 receptors are also expressed in endothelial cells [3,4] within which the presence of included viral bodies has been shown. This determines the accumulation of inflammatory cells and the recruitment of immune cells, both through direct viral invasion of the endothelium and through an immune-mediated mechanism, promoting widespread endothelial dysfunction associated to apoptosis. Therefore, SARS-CoV-2 infection can induce an ‘endothelitis’ in various organs both as a direct consequence of the viral invasion of the endothelium itself and following the inflammatory response of the host [5]. COVID-19 endothelitis could explain the functional impairment of the microcirculation at the systemic level of different vascular districts and the related clinical consequences. The vascular endothelium is, in fact, an indispensable organ for the regulation of vascular tone and for the maintenance of vascular homeostasis [6]. The presence of endothelial damage can promote greater vasoconstriction with consequent organ ischemia, inflammation, tissue edema and a procoagulative state [7] resulting in an increase in the incidence of cardio- and cerebrovascular events. This could be particularly relevant in presence of cardiovascular risk factors (cigarette smoking, high blood pressure, diabetes mellitus, obesity) and/or previous cardiovascular disease with pre-existing endothelial dysfunction: all these risk factors are associated with unfavorable outcome in patients suffering from COVID-19.

Previous treatment might also affect prognosis, in particular it was postulated that ACE inhibitors (ACE-I) could act as a potential risk factor for fatal COVID-19 by up-regulating ACE2 [8,9]. However, there is enough evidence that allows stating also the opposite hypothesis [9,10]. Indeed, there is currently no data and statistics evidencing a direct link between ACE2 activity and SARS-CoV-2 associated mortality or between ACE-I or angiotensin-receptor blockers (ARB) treatment and impaired outcome in COVID-19 [11–13].

Furthermore, the severity of the lung involvement may be relevant from a prognostic point of view. The Brixia score is a radiologic score proposed by Borghesi and Maroldi [14] to assess severity of SARS-CoV-2 pneumonia. However, its prognostic meaning was only demonstrated in a single study [15] and was never assessed in association with other potential predictors of in-hospital mortality.

For all these reasons we considered worthwhile to simultaneously assess several potential predictors of outcome (co-morbidity, previous and in-hospital treatment, Brixia score) in a relatively large population of patients with interstitial pneumonia and respiratory failure due to SARS-CoV-2 related infection.

## Patients and methods

### Patients

We conducted a retrospective cohort study at the Montichiari Hospital, a tertiary health-care centre in Brescia, Italy, which was designated as a COVID-19 hub by the Italian health authorities.

Patients consecutively admitted to the Medicine ward (COVID-19 M unit) of the Montichiari Hospital from February 28 to April 3, 2020 were retrospectively included in the study if they met the following inclusion criteria: (1) confirmed COVID-19 infection as determined by a positive reverse-transcriptase-polymerase-chain-reaction (RT-PCR) assay of a specimen collected on a nasopharyngeal swab; (2) bilateral pulmonary interstitial opacities on chest imaging that were not fully explained by congestive heart failure or other forms of volume overload; (3) an acute respiratory distress syndrome, showing at least one of the following conditions: respiratory rate ≥ 30 breaths/min; peripheral capillary oxygen saturation (SpO_2_) ≤ 93% while breathing ambient air or ratio of the partial pressure of oxygen in arterial blood to the fractional concentration of oxygen in inspired air (PaO_2_/FiO_2_) ≤ 300 mmHg. Patients with critical respiratory syndrome requiring mechanical or invasive ventilation at the admission to our ward were excluded.

This retrospective study has been conducted in accordance with the declaration of Helsinki and its later amendments and was approved by the Ethical Committee of the Spedali Civili of Brescia.

The majority of patients admitted to our Division of Medicine underwent standard therapy (hydroxychloroquine 400 mg daily, lopinavir 800 mg daily plus ritonavir 200 mg per day) [16–18] according to indications of our Institution at that time. Some patients could not be treated with antiviral or antimalarial agents because such drugs were not always available due to an excessive requests from hospitals. In a subgroup of patients with severe respiratory distress intravenous corticosteroids (dexamethasone 20 mg intravenously - i.v. per day for 5 days and then 10 mg i.v. per day for 5 days) were used [19], while another subgroup received off-label a single low dose of the anti-interleukin-6 monoclonal antibody tocilizumab (8 mg/kg mg i.v. - or 324 mg subcutaneously -s.c.- depending on the drug availability) in addition to standard therapy [20]. During hospitalization, patients in both groups were assisted with non-invasive (i.e. low flow nasal cannula; high flow mask; Continuous Positive Airway Pressure -CPAP-) or invasive (i.e. mechanical ventilation) oxygen therapy, according to their needs. Patients were followed until the end of the clinical observation, defined as death or complete recovery and discharge from the hospital with SpO_2_>94% while breathing in ambient air. Starting from the middle of March 2020 some evidence suggesting the possible role of the formation of micro- and macrothrombi was progressively available, together with the evidence of an increased incidence of pulmonary thromboembolism [21–23]; therefore, low-molecular weight heparin (enoxaparin) was empirically administered at different doses. At the beginning, we used a prophylactic dose of 4000 Units s.c. once daily; then, higher doses were progressively used: 4000 Units twice daily, 6000 Units s.c. once daily or 100 Units/kg twice daily (anticoagulation dose), shifting after few days, when indicated, to one of the new oral anticoagulants (apixaban or edoxaban).

The clinical record of each patient was retrospectively analyzed, and, for each patient, the following information was extracted and recorded in a dedicated database: age at admission; gender; comorbidities; date of first flu symptoms; previous drug treatment.

The following laboratory parameters were also evaluated at hospital admission, at the time of discharge and at the time of follow-up circulating white blood cells, granulocytes, monocytes, lymphocytes and hemoglobin, c-reactive protein (CRP), procalcitonin, ferritin, D-dimer, international normalized ratio (INR) and activated partial thromboplastin time (aPTT), plasma creatinine and electrolytes, aspartate aminotransferase (AST), alanine aminotransferase (ALT), and gamma glutamyl transferase (γGT).

In addition, the following ventilator parameters were assessed at admission: SpO_2_%: peripheral capillary oxygen saturation (pulse-oximetry); FiO2: fractional concentration of oxygen in inspired air (i.e. low flow nasal cannula; high flow mask; Continuous Positive Airway Pressure - CPAP). Parameters derived from the first arterial blood gas test performed in the emergency ward: apH: arterial pH; apO_2_: arterial oxygen partial pressure, apCO_2_: arterial carbon dioxide partial pressure; aSpO_2_%: arterial oxygen saturation of hemoglobin; aFiO_2_%: oxygen flow at the moment of the arterial blood gas test; pO_2_/FiO_2_: ratio between arterial oxygen partial pressure and fractional concentration of oxygen in inspired air.

In 155 patients the radiological Brixia score was evaluated, according to Borghesi and Maroldi [14].

Briefly, a score from 0 to 3 is assigned to each of previously defined pulmonary zones based on the lung abnormalities detected on frontal chest projection as follows: score 0 (zero): no lung abnormalities; score 1: interstitial infiltrates, score 2: interstitial and alveolar infiltrates (interstitial predominance), score 3: interstitial and alveolar infiltrates (alveolar predominance).

The scores of the six lung zones are then added together to obtain an overall score ranging from 0 to 18.

Fifty-nine patients died during the hospital admission. All surviving patients were re-evaluated after discharge in July–August 2020.

Outcomes were assessed either at the end of the period of admission to hospital (COVID-19 related death or complete recovery and discharge from the hospital), or after 2–3 months on average (re-evaluation with follow up). Six patients discharged died during the follow up period due to re-aggravation of the pulmonary disease.

### Statistical analyses

Data were analyzed with SPSS version 25.0 (Chicago, IL, U.S.A.). We report categorical variables as number (%) and continuous variables as mean (standard deviation) when data were normally distributed, and as median and interquartile range when data were not normally distributed (Lymphocytes, Procalcitonin, Ferritin, D-dimer). Statistical significance was assessed by means of chi-square test for dichotomous variables, or by means of the two independent sample *t*-test or by Mann–Whitney *U* test when appropriate. A *P*-value <0.05 was considered as statistically significant.

The primary endpoint was the survival rate in the different subgroups. The survival rate was assessed by Kaplan–Meier (KM) plot using subgroups as between factor; death as event and time to death/discharge or time to death/re-evaluation at follow up as time variable. Differences between survival curves were evaluated by Log Rank (Mantel - Cox), Breslow (Generalized Wilcoxon) and Tarone–Ware tests.

The relative importance of prognostic factors at baseline was evaluated in the whole population, and the association of those variables with death events was assessed by the Cox proportional hazard model.

## Results

A total of 258 patients were included in the current study. Sixty-five deaths were observed (59 during admission and 6 after discharge, during the follow-up period). Baseline demographic and clinical characteristics of patients subdivided in dead and survivors are reported in Table 1. Laboratory parameters are reported in Table 2.

**Table 1:** Demographic and clinical characteristics of the two groups of patients

	Dead (*n*=65)	Alive (*n*=193)	All patients (*n*=258)
Age (years)	80.1 ± 7.57***	68.6 ± 19.7	71.0 ± 13.8
Gender (males)	49/65***	124/193	173/258
Diabetes (yes)	23/65***	44/193	67/258
Hypertension (yes)	54/65***	107/193	151/258
COPD (yes)	15/65***	20/193	35/258
Smoke (yes) (actual or previous)	10/65***	30/193	40/258
Obesity (yes)	9/33***	31/145	40/178
Cancer (yes)	7/65***	5/193	12/258
Time of symptoms before hospitalization (days)	5.75 ± 3.58^**^	8.60 ± 8.83	7.92 ± 5.51
*Previous treatment*			
ACE-inhibitors (yes)	21/65***	32/193	53/258
Angiotensin-receptor blockers (yes)	14/65***	40/193	54/258
Statins (yes)	26/65***	53/193	79/258
Anti-platelets agents (yes)	27/65***	48/193	75/258
Steroids (yes)	5/65***	6/193	11/258
Anticoagulants (yes)	11/65***	18/193	29/258

** *P*<0.01 vs. alive; ***P<0.001 v. alive .

**Table 2:** Laboratory parameters of the two groups of patients

	Dead (*n*=65)	Alive (*n*=193)	All patients (*n*=258)
*Humoral parameters:*			
White blood cells (#/mm^3^)	8002 ± 4499 ‡	6315 ± 2936	6740 ± 3467
Granulocytes (#/mm^3^)	6255 ± 4023 ‡	4704 ± 2794	5063 ± 3181
Monocytes (#/mm^3^)	429 ± 376	485 ± 578	472 ± 538
Lymphocytes (#/mm^3^)	835 (663)	910 (550)	880(525)
Hemoglobin (g/dl)	14.1 ± 12.1	12.8 ± 1.69	13.1 ± 6.26
CRP (mg/l)	132 ± 85.6 ‡	88.2 ± 21.4	99.2 ± 77.1
Procalcitonin (ng/ml)	0.14(0.11)†	0.12(0.26)	0.12(0.21)
Ferritin (µg/l)	636(247)	9696(1029)	653(739)
D-dimer (ng/ml)	425(995)	502(882)	447(847)
INR	1.336 ± 0.287	1.423 ± 2.240	1.40 ± 1.94
aPTT (sec)	32.5 ± 3.58	32.7 ± 4.09	32.7 ± 3.96
*Ventilatory parameters:*			
SpO_2_%	92.3 ± 5.80 ‡	95.2 ± 2.24	94.5 ± 3.80
FiO_2_	51.7 ± 32.5 ‡	36.0 ± 19.9	40.0 ± 24.6
apH	7.47 ± 0.066 †	7.49 ± 0.048	7.48 ± 0.054
apO_2_ (mm Hg)	52.5 ± 19.3 ‡	63.0 ± 16.7	60.3 ± 17.9
apCO_2_ (mm Hg)	33.6 ± 8.75	34.8 ± 5.45	34.5 ± 6.46
aSpO_2_%	82.6 ± 15.1 ‡	91.5 ± 6.71	89.2 ± 10.3
aFiO_2_%	27.4 ± 16.7	24.4 ± 12.3	25.1 ± 13.6
pO_2_/FiO_2_	220 ± 86.6 ‡	281 ± 83.1	265 ± 87.9

CRP: C-reactive protein; INR: international normalized ratio, aPTT: activated partial thromboplastin time; SpO_2_%: peripheral capillary oxygen saturation (pulse-oximetry); FiO2: fractional concentration of oxygen in inspired air (i.e. low flow nasal cannula; high flow mask; Continuous Positive Airway Pressure - CPAP). Parameters derived from the first arterial blood gas test performed in the emergency ward: apH: arterial pH; apO2: arterial oxygen partial pressure, arterial carbon dioxide partial pressure; aSpO2%: arterial oxygen saturation of hemoglobin; aFiO2%: oxygen flow at the moment of the arterial blood gas test; pO2/FiO2: ratio between arterial oxygen partial pressure and fractional concentration of oxygen in inspired air.

Paired *t*-test or Mann–Whitney *U* test: †*P*<0.01, ‡*P*<0.001 vs. alive.

### Brixia score

The radiologic Brixia score was significantly correlated with some ventilatory and inflammatory indices, in particular FiO_2_, apO_2_, aSpO_2_%, pO_2_/FiO_2_ and CRP (Table 3).

**Table 3:** Correlations between Brixia radiologic score and ventilatory or inflammatory indices (univariate analysis)

Brixia score and:	***r***	***P***
SpO_2_%	-0.176	0.034
FiO_2_	0.366	<0.001
apH	0.029	NS
apO_2_	-0.410	<0.001
apCO_2_	-0.006	NS
aSpO_2_%	-0.36	<0.001
aFiO_2_%	0.142	0.097
pO_2_/FiO_2_	-0.465	<0.001
White blood cells	0.188	0.023
Granulocytes	0.206	0.016
CRP	0.386	<0.001
Ferritin serum levels	0.319	0.011
Procalcitonin	-0.043	NS

SpO_2_%: peripheral capillary oxygen saturation (pulse-oximetry); FiO_2_: fractional concentration of oxygen in inspired air (i.e. low flow nasal cannula; high flow mask; Continuous Positive Airway Pressure -CPAP). Parameters derived from the first arterial blood gas test performed in the emergency ward: apH: arterial pH; apO_2_: arterial oxygen partial pressure, arterial carbon dioxide partial pressure; aSpO_2_%: arterial oxygen saturation of hemoglobin; aFiO_2_%: oxygen flow at the moment of the arterial blood gas test; pO_2_/FiO_2_: ratio between arterial oxygen partial pressure and fractional concentration of oxygen in inspired air; CRP: C-reactive protein; NS: not statistically significant.

**Figure 1:**
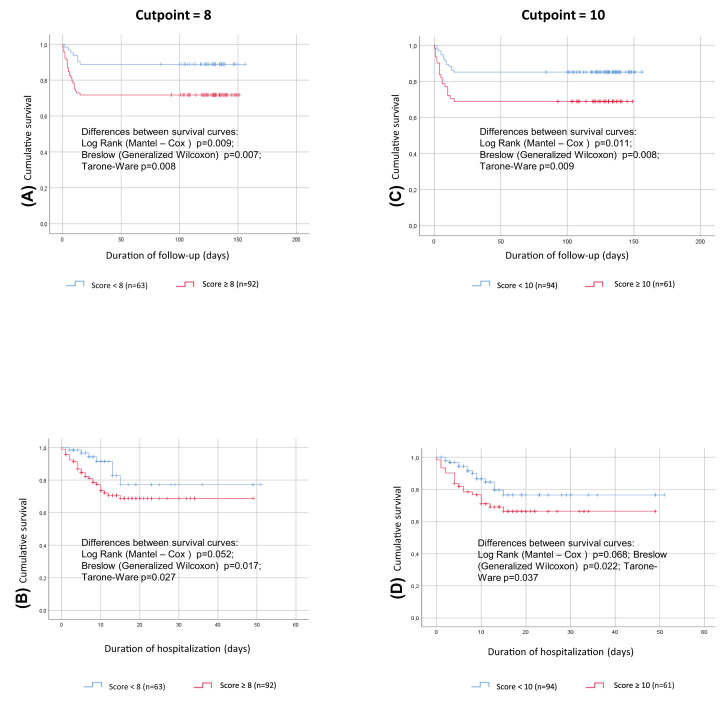
Kaplan–Meier survival curve for the Brixia radiologic score Analysis run using group as factor; death as event and time to death/discharge or time to death/re-evaluation at follow up as time variable. (**A**) Brixia score ≥ 8 (red line) or < 8 (blue line), time to death/re-evaluation at follow up as time variable, (**B**) Brixia score ≥ 8 (red line) or < 8 (blue line), time to death/discharge as time variable, (**C**) Brixia score ≥ 10 (red line) or < 8 (blue line), time to death/re-evaluation at follow up as time variable, (**D**) Brixia score ≥ 10 (red line) or < 8 (blue line), time to death/discharge as time variable.

We subdivided patients according to the presence of a Brixia score < or ≥ 8, a cutpoint previously demonstrated to be clinically relevant [15]. A significant worsening of survival was clearly observed (Figure 1), both when the whole period of follow up or just the period of admission to the hospital were considered. When using a more conservative cutpoint (< or ≥ 10) the survival curves were substantially similar.

### Cardiovascular risk factors and previous treatments

**Figure 2:**
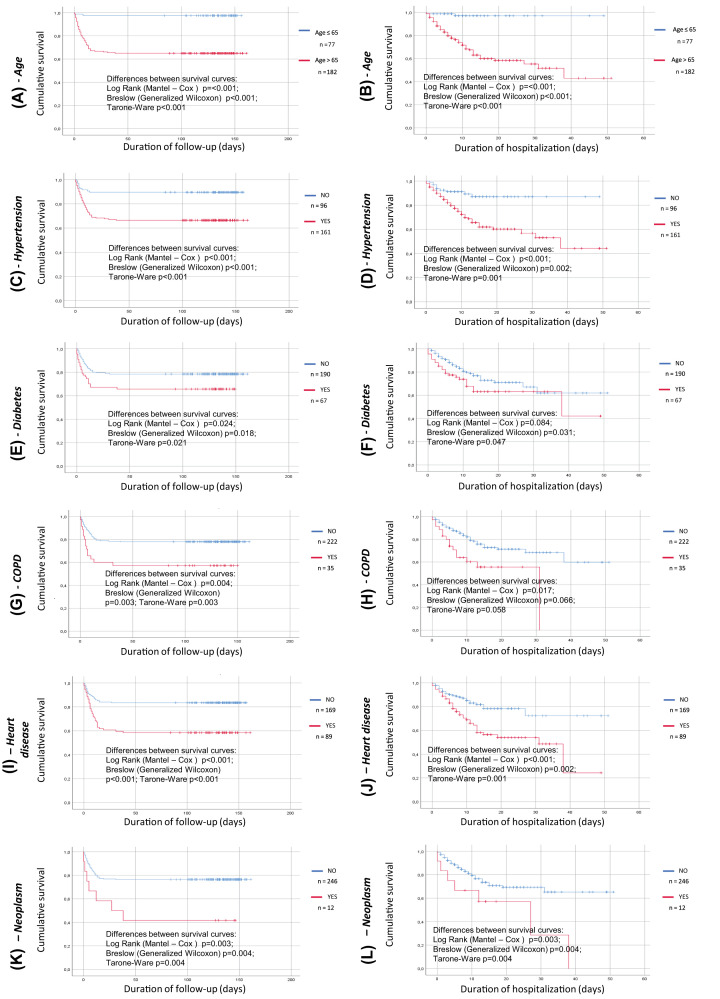
Kaplan–Meier survival curve for the presence or absence of cardiovascular risk factors or comorbidities Analysis run using group as factor; death as event and time to death/discharge or time to death/re-evaluation at follow up as time variable. (**A**) age ≥ 65 (red line) or < 65 (blue line), time to death/re-evaluation at follow up as time variable, (**B**) age ≥ 65 (red line) or < 65 (blue line), time to death/discharge as time variable, (**C**) presence (red line) or absence (blue line) of hypertension at entry, time to death/re-evaluation at follow up as time variable, (**D**) presence (red line) or absence (blue line) of hypertension at entry, time to death/discharge as time variable, (**E**) presence (red line) or absence (blue line) of diabetes mellitus at entry, time to death/re-evaluation at follow up as time variable, (**F**) presence (red line) or absence (blue line) of diabetes mellitus at entry, time to death/discharge as time variable, (**G**) presence (red line) or absence (blue line) of chronic obstructive pulmonary disease at entry, time to death/re-evaluation at follow up as time variable, (**H**) presence (red line) or absence (blue line) of chronic obstructive pulmonary disease at entry, time to death/discharge as time variable, (**I**) presence (red line) or absence (blue line) of any cardiac disease at entry, time to death/re-evaluation at follow up as time variable, (**J**) presence (red line) or absence (blue line) of any cardiac disease at entry, time to death/discharge as time variable, (**K**) presence (red line) or absence (blue line) of any active tumor entry at entry, time to death/re-evaluation at follow up as time variable, (**L**) presence (red line) or absence (blue line) of any active tumor entry, time to death/discharge as time variable

Patients were subdivided according to the presence of arterial hypertension, chronic obstructive pulmonary disease (COPD), diabetes mellitus, cardiac disease, tumors and age <65 years. In all cases, survival was significantly reduced when such factors or comorbidities were present (Figure 2), both when the whole period of follow up or just the period of admission to the hospital were considered, apart from a borderline statistical significance for the presence of cancer when the longer follow up period was considered.

In our statistical analysis, smoking habit, obesity and gender were not predictors of outcome, or the difference between curves did not reach statistical significance.

**Figure 3:**
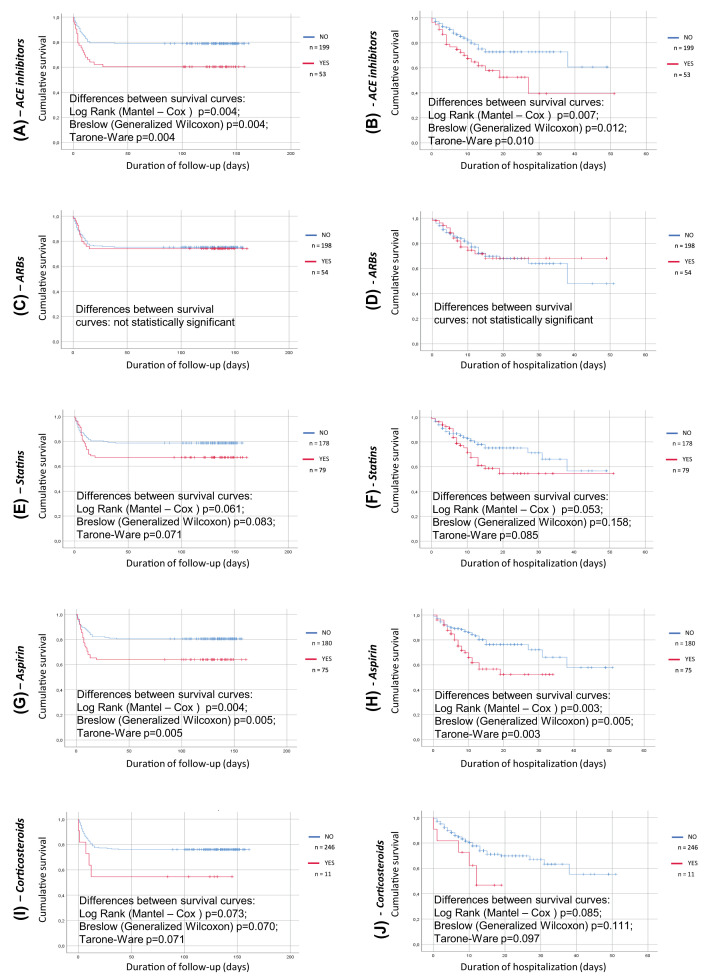
Kaplan–Meier survival curve for pre-admission chronic treatments Analysis run using group as factor; death as event and time to death/discharge or time to death/re-evaluation at follow up as time variable. (**A**) previous treatment with ACE-inhibitors (red line) or no treatment with ACE-inhibitors (blue line), time to death/re-evaluation at follow up as time variable, (**B**) previous treatment with ACE-inhibitors (red line) or no treatment with ACE-inhibitors (blue line), time to death/discharge as time variable, (**C**) previous treatment with angiotensin-receptor blockers (red line) or no treatment with angiotensin-receptor blockers (blue line), time to death/re-evaluation at follow up as time variable, (**D**) previous treatment with angiotensin-receptor blockers (red line) or no treatment with angiotensin-receptor blockers (blue line), time to death/discharge as time variable, (**E**) previous treatment with statins (red line) or no treatment with statins (blue line), time to death/re-evaluation at follow up as time variable, (**F**) previous treatment with statins (red line) or no treatment with statins (blue line), time to death/discharge as time variable, (**G**) previous treatment with anti-platelet drugs (red line) or no treatment with anti-platelet drugs (blue line), time to death/re-evaluation at follow up as time variable, (**H**) previous treatment with anti-platelet drugs (red line) or no treatment with anti-platelet drugs (blue line), time to death/discharge as time variable, (**I**) previous treatment with steroids (red line) or no treatment with steroids (blue line), time to death/re-evaluation at follow up as time variable, (**J**) previous treatment with steroids (red line) or no treatment with steroids (blue line), time to death/discharge as time variable.

When previous drug treatment was considered, patients treated with ACE-I showed a worse survival rate compared with those not taking ACE-I, while survival curves for those treated or not with ARB were superimposable (Figure 3). This was again true considering either the short or the prolonged period of observation. An impaired survival rate was also observed in those patients previously treated with antiplatelet drugs (Figure 3), while previous treatment with anticoagulants, statins or steroids was not associated to an impaired survival in a statistically significant manner (Figure 3).

### Anticoagulant treatment during admission

**Figure 4:**
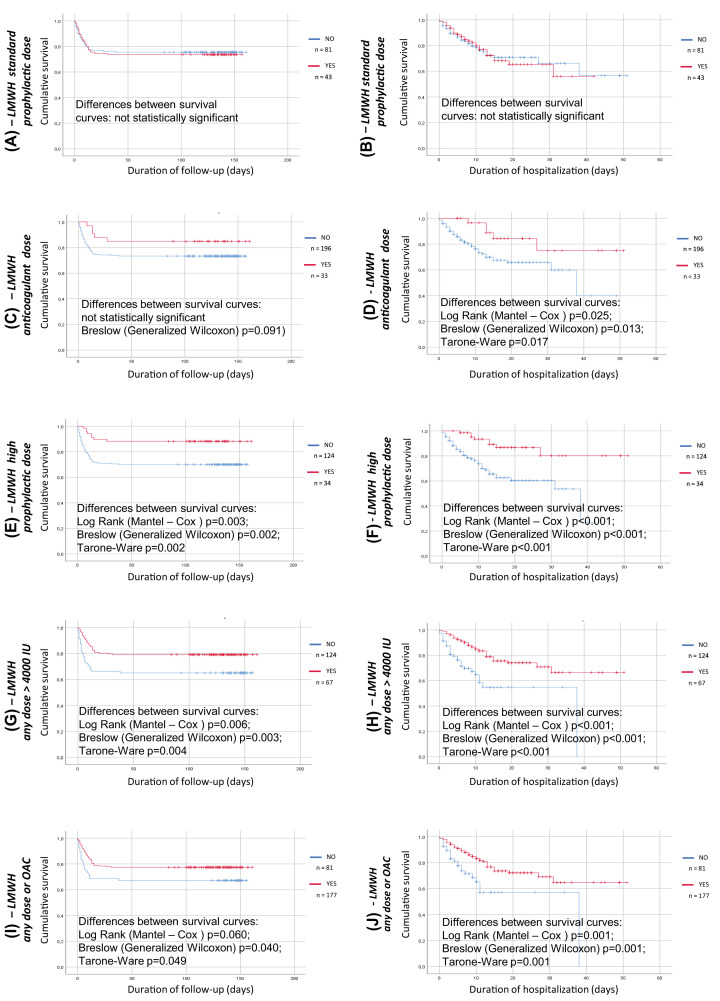
Kaplan–Meier survival curve for the anticoagulant drug treatments during admission Analysis run using group as factor; death as event and time to death/discharge or time to death/re-evaluation at follow up as time variable. (**A**) treatment with enoxaparin 4000 Units subcutaneously once daily (red line) or no anticoagulant treatment (blue line), time to death/re-evaluation at follow up as time variable, (**B**) treatment with enoxaparin 4000 Units subcutaneously once daily (red line) or no anticoagulant treatment (blue line), time to death/discharge as time variable, (**C**) treatment with treatment with enoxaparin 100 Units/kg twice daily (anticoagulation dose) (red line) or no anticoagulant treatment (blue line), time to death/re-evaluation at follow up as time variable, (**D**) treatment with enoxaparin 100 Units/kg twice daily (anticoagulation dose) (red line) or no anticoagulant treatment (blue line), time to death/discharge as time variable, (**E**) treatment with treatment with any dose of enoxaparin greater than 4000 Units subcutaneously once daily (red line) or no anticoagulant treatment (blue line), time to death/re-evaluation at follow up as time variable, (**F**) treatment with any dose of enoxaparin greater than 4000 Units subcutaneously once daily (red line) or no anticoagulant treatment (blue line), time to death/discharge as time variable, (**G**) treatment with treatment with any dose of enoxaparin or no anticoagulant treatment (blue line), time to death/re-evaluation at follow up as time variable, (**H**) treatment with any dose of enoxaparin once daily (red line) or no anticoagulant treatment (blue line), time to death/discharge as time variable, (**I**) treatment with treatment with any dose of enoxaparin ±any new oral direct anticoagulant or no anticoagulant treatment (blue line), time to death/re-evaluation at follow up as time variable, (**J**) treatment with any dose of enoxaparin once daily (red line) ± any new oral direct anticoagulant or no anticoagulant treatment (blue line), time to death/discharge as time variable.

While the administration of enoxaparin at low, prophylactic dose (4000 Units s.c. once daily) was not associated with any effect on survival(Figure 4), higher doses (4000 Units twice daily, 6000 Units s.c. once daily or 100 Units/kg twice daily (anticoagulant dose), when considered together, significantly improved survival (Figure 4) considering either the short or the prolonged period of observation. Also the combination of enoxaparin and new oral direct anticoagulants was effective in this regard (Figure 4). The survival curves tend to diverge progressively while increasing the extent of anticoagulation. The effect on survival of the different doses of enoxaparin is reported in the online Supplementary Figure 1, while the effects on survival of other treatment strategies adopted in our ward are reported in the online Supplementary Figure 2.

### Multivariate Cox regression analysis

The relative importance of prognostic factors at baseline, such as age, gender, smoking habit, presence of arterial hypertension, obesity, diabetes, chronic obstructive pulmonary disease, active tumors, cardiac disease and previous drug treatment (ACEI, ARB, antiplatelet drugs, statins, steroids, anticoagulants) was evaluated in the whole population, and the association of those variables with death events was assessed by the Cox proportional hazard model considering the period of admission to the hospital. Only age (*P*<0.001), the presence of chronic obstructive pulmonary disease (*P*=0.003) and previous treatment with ACEI (*P*=0.002) or steroids (*P*=0.041) remained in the model and were found to be significantly and independently associated with the occurrence of death.

The analysis was repeated including the Brixia radiologic score (as a continuous variable or with the two cutpoints 8 and 10); however the variable did not enter the model (Table 4).

**Table 4:** Multivariate Cox regression analysis performed on the following variables: age, gender, smoking status, presence of arterial hypertension, obesity, diabetes, chronic obstructive pulmonary disease, active tumors, cardiac disease and previous drug treatment (ACEI, ARB, antiplatelet drugs, statins, steroids, anticoagulants**;** Brixia radiologic score as a categorical variable (outpoint 8)

	Variables that enter the equation					
	B	SE	Wald	gl	Sign.	Exp(*B*)
Step 1						
Age	0.113	0.034	11,243	1	0.001	1.120
Step 2						
Age	0.137	0.041	11,075	1	0.001	1.147
Previous treatment with ACE inhibitors	-1.240	0.603	4,226	1	0.040	0.289
Step 3						
Age	0.143	0.045	10,086	1	0.001	1.154
Chronic obstructive pulmonary disease	-1.489	0.643	5,359	1	0.021	0.226
Previous treatment with ACE inhibitors	-1.868	0.727	6,605	1	0.010	0.154
Step 4						
Age	0.144	0.048	9,157	1	0.002	1.155
Chronic obstructive pulmonary disease	-1.792	0.691	6,732	1	0.009	0.167
Previous treatment with ACE inhibitors	-2.235	0.797	7,870	1	0.005	0.107
Previous treatment with Steroids	-1.911	0.872	4,796	1	0.029	0.148

When all the previously mentioned variables were considered together with treatments with various doses of enoxaparin, age (*P*<0.001) and treatment with enoxaparin at a dose higher than 4000 Units (*P*=0.05) entered the model. Finally, were included in the analysis, together with the previously mentioned variables (cardiovascular risk factors, comorbidities, previous treatments, treatment with enoxaparin during admission, Brixia score), also the following ventilator parameters: SpO_2_%, FiO_2_, apH, apO_2_, apCO_2_, aSpO_2_%, aFiO_2_% and pO_2_/FiO_2_, as well as humoral variables (CRP, procalcitonin, number of circulating white blood cells and granulocytes), the following variables entered the model: procalcitonin circulating levels (*P*<0.003), FiO_2_ (*P*=0.010) and granulocytes (*P*=0.02) Similar results were obtained if the prolonged period of observation (until follow up) was considered.

## Discussion

Severe acute respiratory syndrome coronavirus 2 (SARS-CoV-2) has been characterized by a very high mortality rate being interstitial pneumonia with respiratory failure the main cause of death for COVID-19 [1]. Among COVID-19 patients, about 25% present a severe complication of infection including acute respiratory distress syndrome (ARDS), with a rapid worsening of clinical conditions leading to the need of mechanical or invasive ventilation to support respiratory functions in intensive care units [24]. Although the direct cytopathic effect of the virus and viral invasion seems to be critical for a worsening of the clinical course, there is evidence that other factors may be implicated in the prognosis of the acute respiratory distress syndrome [25].

Furthermore, in accordance with previous data [26], the current study also supports the concept that older age is a risk factor for death. Several reports also suggested that concomitant chronic illnesses may have an impact on mortality rate [1,27]. Our study further supports that diabetes and underlying cardiac disease are risk factors for worsening outcome, having patients with diabetes or cardiac disease a worse survival in respect with patients without diabetes or without cardiac disease [28,29]. In our cohort also the presence of hypertension was associated with a worse outcome [28,29]. The role of older age (>65 years), cardiac disease, chronic obstructive pulmonary disease as well as of smoking was also confirmed in a large study (*n*=8910) by Mehra MR et al. [Cardiovascular disease, drug therapy, and mortality in covid-19 disease. N Engl J Med. 2020 Jun 18;382(25):e102], that was, however, subsequently retracted because all the authors were not granted access to the raw data and the raw data could not be made available to a third-party auditor.

Surprisingly, in our study previous treatment with ACEI but not with ARB was associated with a worse survival. Although, as mentioned, there are theoretical reasons to postulate that treatment with ACEI and ARB might increase expression of ACE2, thus favoring viral interaction with target cells [3,4,8,9] some studies failed to demonstrate an impaired outcome in patients treated with blockers of the renin-angiotensin system [11–13,30,31]. An interaction may exist in patients with COVID-19 between comorbidities including hypertension, diabetes and established cardiovascular disease and treatment with different antihypertensive drug classes [32,33].

A very recent study from the SARS-RAS Study of the Italian Society of Hypertension, performed in 1591 patients addressed in detail the problem of possible prognostic role of hypertension and hypertension-related treatment with renin-angiotensin system inhibitors, also considering possible confounders [34]. In nonsurvivors, older age, hypertension, diabetes mellitus, chronic obstructive pulmonary disease, chronic kidney disease, coronary artery diseases, and heart failure were more represented than in survivors [34]. The Charlson Comorbidity Index was significantly higher in nonsurvivors compared with survivors [34]. ACEI (but not ARB), diuretics, and β-blockers were more frequently used in nonsurvivors than in survivors, but this could be due to a worse clinical profile of patients treated with these drugs in terms of comorbidities and cardiovascular risk factors [34]. In fact, in the abovementioned study, ACE inhibitors lose their statistical significance when corrected by comorbidities and age [34]. Also, in our study patients treated with ACEI were older and had a greater prevalence of cardiac disease.

A very interesting result of our study is the observation of a prognostic role of the Brixia radiologic score [14], confirming a previous observation [15]. Our data support what previously observed according to receiver operating characteristic curve analyses that the optimal cutoff values for Brixia score was 8 points. Therefore, the Brixia score showed an excellent predictive power, and this was recently acknowledged as a good tool for stratification of risk in a document of the WHO [35]. According to our Multivariate Cox regression analysis some of the information carried by the Brixia score is also carried by the ventilator parameters (oxygen saturation, oxygen flow, etc.), as well as by cardiovascular risk factors. However, the assessment of the Brixia radiologic score in a COVID-19 patient admitted to a ward might provide a quick and comprehensive indicator of the severity of the disease, and may be therefore considered a useful prognostic tool.

Interestingly enough, in our study treatment with the low-molecular weight heparin enoxaparin was demonstrated to have a positive impact on survival only when given at doses greater than the prophylactic dose (4000 Units once daily). Anticoagulant treatment was demonstrated to be associated with decreased mortality in severe coronavirus disease 2019 patients with coagulopathy [36]. Our results support the administration of anticoagulant drugs at proper doses in all COVID-19 patients, as also suggested by some guidelines [37–38]. The possible beneficial, harmful or neutral role of ACEI or ARB remains a matter of debate, and larger studies are probably needed [39,40].

In conclusion, despite the limitations of the current study, mainly due to its retrospective nature, the results are clearly relevant since they demonstrate that some co-morbidities and cardiovascular risk factors may affect prognosis. The radiologic Brixia score may be a useful tool for stratifying the risk of death at baseline, and anticoagulant treatment might be associated to a clinical benefit in terms of survival in patients with COVID-19.

## Perspectives

- The aim of the study was to assess simultaneously several potential predictors of outcome (co-morbidity, previous and in-hospital treatment, radiologic Brixia score) in patients with COVID-19.
- Some co-morbidities and cardiovascular risk factors may affect prognosis. The radiologic Brixia score may be a useful tool for stratifying the risk of death at baseline, and some treatments, in particular anticoagulant treatment, might be associated to a clinical benefit in terms of survival in patients with COVID-19.
- The observation that the administration of enoxaparin at the low, prophylactic dose of 4000 Units per day was ineffective on survival may be of clinical interest for the treatment of COVID-19.

## Supplementary Material

Supplementary Figures S1-S2

## Data Availability

Original data will be provided upon request after removal of personal identifiers.

## Abbreviations

γGT: gamma glutamyl transferase
ALT: alanine aminotransferase
aPTT: activated partial thromboplastin time
ARDS: acute respiratory distress syndrome
AST: aspartate aminotransferase
CRP: c-reactive protein
INR: international normalized ratio

## References

[1] Guan W.J., Ni Z.Y., Hu Y., Liang W.H., Ou C.Q., He J.X. et al. (2020) China Medical Treatment Expert Group for Covid-19. Clinical characteristics of coronavirus disease 2019 in China. N. Engl. J. Med. 382, 1708–1720 10.1056/NEJMoa2002032 32109013 PMC7092819

[2] Badawi A. and Ryoo S.G. (2016) Prevalence of comorbidities in the Middle East respiratory syndrome coronavirus (MERS-CoV): a systematic review and meta-analysis. Int. J. Infect. Dis. 49, 129–133 10.1016/j.ijid.2016.06.015 27352628 PMC7110556

[3] Ferrario C.M., Jessup J., Chappell M.C. et al. (2005) Effect of angiotensin-converting enzyme inhibition and angiotensin II receptor blockers on cardiac angiotensin-converting enzyme 2. Circulation 111, 2605–2610 10.1161/CIRCULATIONAHA.104.510461 15897343

[4] Monteil V K.H., Prado P., Hagelkrüys A. et al. (2020) Inhibition of SARS-CoV-2 infections in engineered human tissues using clinical-grade soluble human ACE2. Cell 181, 905–913 10.1016/j.cell.2020.04.004 32333836 PMC7181998

[5] Varga Z., Flammer A.J., Steiger P., Haberecker M., Andermatt R., Zinkernagel A.S. et al. (2020) Endothelial cell infection and endotheliitis in COVID-19. Lancet 395, 1417–1418 10.1016/S0140-6736(20)30937-5 32325026 PMC7172722

[6] Flammer A.J., Anderson T., Celermajer D.S. et al. (2012) The assessment of endothelial function: from research 168-75 into clinical practice. Circulation 126, 753–67 10.1161/CIRCULATIONAHA.112.093245 22869857 PMC3427943

[7] Bonetti P.O., Lerman L.O. and Lerman A. (2003) Endothelial dysfunction - a marker of atherosclerotic risk. Arterioscl. Throm. Vasc. Biol. 23, 168–175 10.1161/01.ATV.0000051384.43104.FC 12588755

[8] Hypothesis D.J.H. (2020) angiotensin-converting enzyme inhibitors and angiotensin receptor blockers may increase the risk of severe COVID-19. J. Travel Med. 27, 041 10.1093/jtm/taaa041 PMC7184445 32186711

[9] Sommerstein R., Kochen M.M., Messerli F.H. and Ch Gräni (2020) Coronavirus Disease 2019 (COVID-19): do angiotensin-converting enzyme inhibitors/angiotensin receptor blockers have a biphasic effect? J. Am. Heart Assoc. 9, e016509 10.1161/JAHA.120.016509 32233753 PMC7428596

[10] Ruilope L.M., Tamargo J. and Ruiz-Hurtado G. (2020) Renin-angiotensin system inhibitors in the COVID-19 pandemic: consequences of antihypertensive drugs. Eur. Heart J. 41, 2067–2069 10.1093/eurheartj/ehaa487 32498078 PMC7314098

[11] Gao C., Cai Y., Zhang K. et al. (2020) Association of hypertension and antihypertensive treatment with COVID-19 mortality: a retrospective observational study. Eur. Heart J. 41, 2058–2066 10.1093/eurheartj/ehaa433 32498076 PMC7314067

[12] Schiffrin E.L., Flack J.M., Ito S., Muntner P. and Webb R.C. (2020) Hypertension and COVID-19. Am. J. Hypertens. 33, 373–374 10.1093/ajh/hpaa057 32251498 PMC7184512

[13] Kuster G.M., Pfister O., Burkard T., Zhou Q., Twerenbold R. et al. (2020) 558 SARS-CoV2: should inhibitors of the renin-angiotensin system be withdrawn in patients with COVID-19? Eur. Heart J. 41, 1801–1803 10.1093/eurheartj/ehaa235 32196087 PMC7184407

[14] Borghesi A. and Maroldi R. (2020) COVID-19 outbreak in Italy: experimental chest X-ray scoring system for quantifying and monitoring disease progression. Radiol. Med. 1–5 32358689 10.1007/s11547-020-01200-3 PMC7194501

[15] Borghesi A., Zigliani A., Golemi S., Carapella N., Maculotti P., Farina D. et al. (2020) Chest X-ray severity index as a predictor of in-hospital mortality in coronavirus disease 2019: A study of 302 patients from Italy. Int. J. Infect. Dis. 96, 291–293 10.1016/j.ijid.2020.05.021 32437939 PMC7207134

[16] Chu C.M., Cheng V.C., Hung I.F. et al. (2004) Role of lopinavir/ritonavir in the treatment of SARS: initial virological and clinical findings. Thorax 59, 252–256 10.1136/thorax.2003.012658 14985565 PMC1746980

[17] Vincent M.J., Bergeron E., Benjannet S. et al. (2005) Chloroquine is a potent inhibitor of SARS coronavirus infection and spread. Virol. J. 2, 69 10.1186/1743-422X-2-69 16115318 PMC1232869

[18] Sanders J.M., Monogue M.L., Jodlowski T.Z. and Cutrell J.B. (2020) Pharmacologic treatments for coronavirus disease 2019 (COVID-19): a review. JAMA 323, 1824–1836 32282022 10.1001/jama.2020.6019

[19] The RECOVERY Collaborative Group (2020) Dexamethasone in hospitalized patients with Covid-19 — Preliminary Report. N. Engl. J. Med. NEJMoa2021436 10.1056/NEJMoa2021436 PMC7383595 32678530

[20] De Rossi N., Scarpazza C., Filippini C., Cordioli C., Rasia S., Mancinelli C.R. et al. (2020) Early use of low dose tocilizumab in patients with COVID-19: A retrospective cohort study with a complete follow-up. EClinicalMedicine 11, 08 10.1016/j.eclinm.2020.100459 PMC7366117 32838235

[21] Zhang Y., Cao W., Xiao M., Li Y.J., Yang Y., Zhao J. et al. (2020) Clinical and coagulation characteristics of 7 patients with critical COVID-2019 pneumonia and acro-ischemia. Zhonghua Xue Ye Xue Za Zhi 41, 302–307 32447934 10.3760/cma.j.issn.0253-2727.2020.0006 PMC7364914

[22] Yin S., Huang M., Li D. and Tang N. (2020) Difference of coagulation features between severe pneumonia induced by SARS-CoV2 and non-SARS-CoV2. J. Thromb. Thrombolysis 1–4 Online ahead of print 10.1007/s11239-020-02105-8 PMC7124128 32246317

[23] Thachil J. (2020) The versatile heparin in COVID-19. J. Thromb. Haemost. 18, 1020–1022 10.1111/jth.14821 32239799 PMC9906146

[24] Huang C., Wang Y., Li X. et al. (2020) Clinical features of patients infected with 2019 novel coronavirus in Wuhan, China. Lancet 395, 497–506 10.1016/S0140-6736(20)30183-5 31986264 PMC7159299

[25] Xu Z., Shi L., Wang Y. et al. (2020) Pathological findings of COVID-19 associated with acute respiratory distress syndrome. Lancet Respir. Med. 8, 420–422 10.1016/S2213-2600(20)30076-X 32085846 PMC7164771

[26] Zhou F., Yu T., Du R. et al. (2020) Clinical course and risk factors for mortality of adult inpatients with COVID-19 in Wuhan, China: a retrospective cohort study. Lancet 395, 1054–1062 10.1016/S0140-6736(20)30566-3 32171076 PMC7270627

[27] Wu Z. and McGoogan J.M. (2020) Characteristics of and important lessons from the coronavirus disease 2019 (COVID-19) outbreak in China: summary of a report of 72314 cases from the Chinese Center for Disease Control and Prevention. JAMA 323, 1239–1242 10.1001/jama.2020.2648 32091533

[28] Cook T.M. (2020) The importance of hypertension as a risk factor for severe illness and mortality in COVID-19. Anaesthesia 75, 976–977 10.1111/anae.15103 32339251 PMC7267452

[29] Jordan R.E., Adab P. and Cheng K.K. (2020) Covid-19: risk factors for severe disease and death. BMJ 368, m1198 10.1136/bmj.m1198 32217618

[30] Sanchis-Gomar F., Lavie C.J., Perez-Quilis C., Henry B.M. and Lippi G. (2020) Angiotensin-Converting Enzyme 2 and antihypertensives (Angiotensin Receptor Blockers and Angiotensin-Converting Enzyme Inhibitors) in coronavirus disease 2019. Mayo Clin. Proc. 95, 1222–1230 10.1016/j.mayocp.2020.03.026 32376099 PMC7129862

[31] Yang G., Tan Z., Zhou L., Yang M., Peng L., Liu J. et al. (2020) Effects of Angiotensin II Receptor Blockers and ACE (Angiotensin-Converting Enzyme) Inhibitors on virus infection, inflammatory status, and clinical outcomes in patients with COVID-19 and hypertension: a single-center retrospective study. Hypertension 76, 51–58 10.1161/HYPERTENSIONAHA.120.15143 32348166

[32] Singh A.K., Gupta R. and Misra A. (2020) Comorbidities in COVID-19: Outcomes in hypertensive cohort and controversies with renin angiotensin system blockers. Diab. Metab. Syndr. 14, 283–287 10.1016/j.dsx.2020.03.016 PMC7144598 32283499

[33] Zhou X., Zhu J. and Xu T. (2020) Clinical characteristics of coronavirus disease 2019 (COVID-19) patients with hypertension on renin-angiotensin system inhibitors. Clin. Exp. Hypertens. 42, 656–660 10.1080/10641963.2020.1764018 32404011 PMC7232880

[34] Iaccarino G., Grassi G., Borghi C., Ferri C., Salvetti M., Volpe M. et al. (2020) Age and multimorbidity predict death among COVID-19 patients: results of the SARS-RAS study of the Italian Society of Hypertension. Hypertension 76, 366–372 10.1161/HYPERTENSIONAHA.120.15324 32564693

[35] World Health Organization Corticosteroids for COVID-19. Living guidance 2 September 2020, page 22. WHO reference number: WHO/2019-nCoV/Corticosteroids/2020.1

[36] Tang N., Bai H., Chen X., Gong J., Li D. and Sun Z. (2020) Anticoagulant treatment is associated with decreased mortality in severe coronavirus disease 2019 patients with coagulopathy. J. Thromb. Haemost. 18, 1094–1099 10.1111/jth.14817 32220112 PMC9906401

[37] World Health Organization Clinical management of COVID-19. Interim guidance 27 May 2020. WHO reference number: WHO/2019-nCoV/clinical/2020.5

[38] COVID-19 Treatment Guidelines Panel Coronavirus Disease 2019 (COVID-19) Treatment Guidelines National Institutes of Health Available at https://www.covid19treatmentguidelines.nih.gov/ Accessed on november 2 2020 34003615

[39] Huang Z., Jiang Y., Chen J. and Zhou Y. (2020) Inhibitors of the renin-angiotensin system: the potential role in the pathogenesis of COVID-19. Cardiol. J. 27, 171–174 10.5603/CJ.a2020.0056 32286678 PMC8016038

[40] Di Castelnuovo A., De Caterina R., de Gaetano G. and Iacoviello L. (2020) Controversial relationship between renin-angiotensin system inhibitors and severity of COVID-19: announcing a large multicentre case-control study in Italy. Hypertension 76, 312–313 10.1161/HYPERTENSIONAHA.120.15370 32383624

