# Supplementary material for: Prognostic factors and predictors of outcome in patients with COVID-19 and related pneumonia: a retrospective cohort study
Source: Biosci Rep. 2020 Dec 4;40(12):BSR20203455. doi: 10.1042/BSR20203455 (PMC7724688; doi:10.1042/BSR20203455)
Supplement: Supplementary Figures S1-S2 [file BSR-2020-3455_supp.pdf]

Figure 1 Supplementary Material – Different doses of LMWH

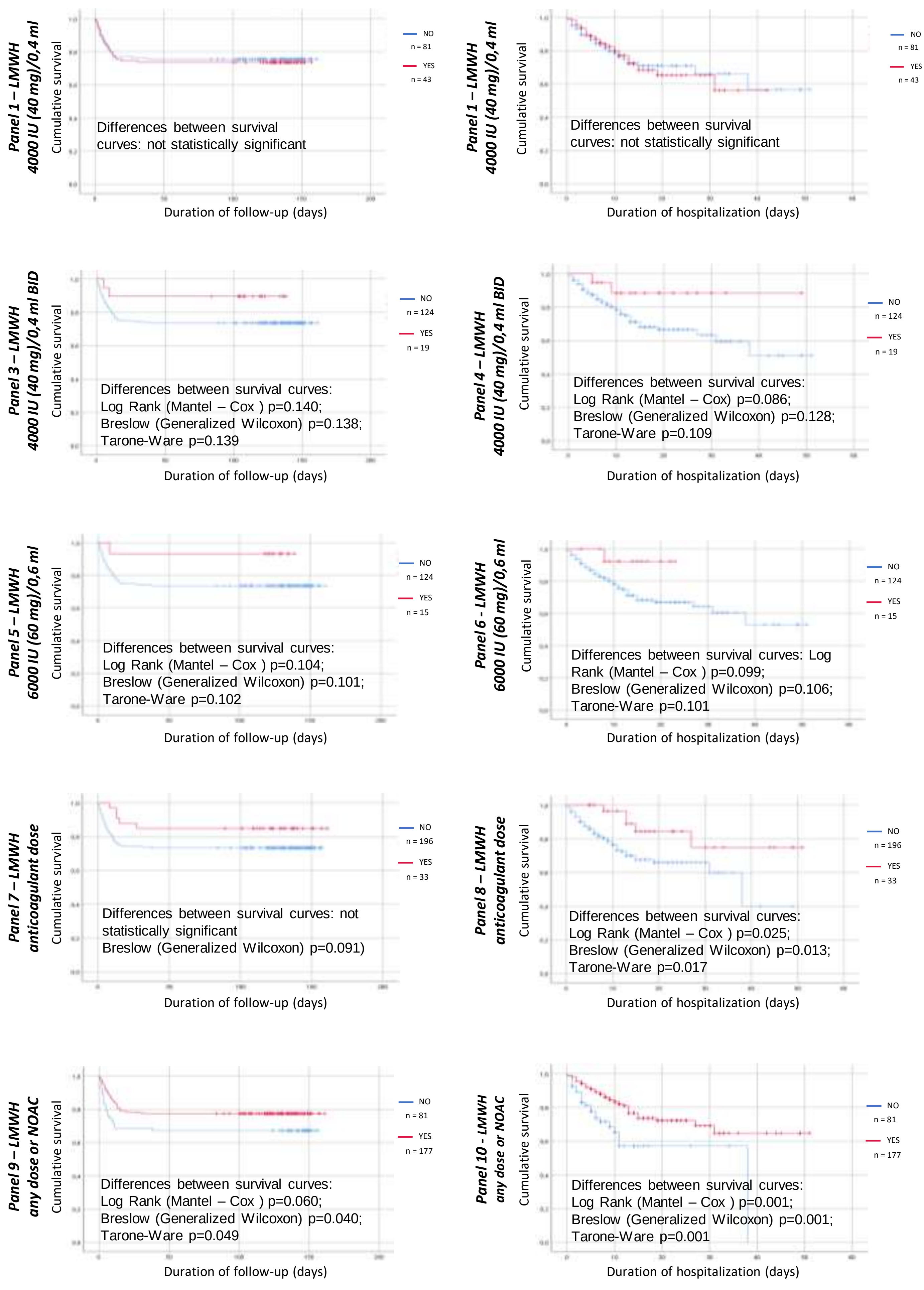

Figure 2 Supplementary Material – Treatments during admission

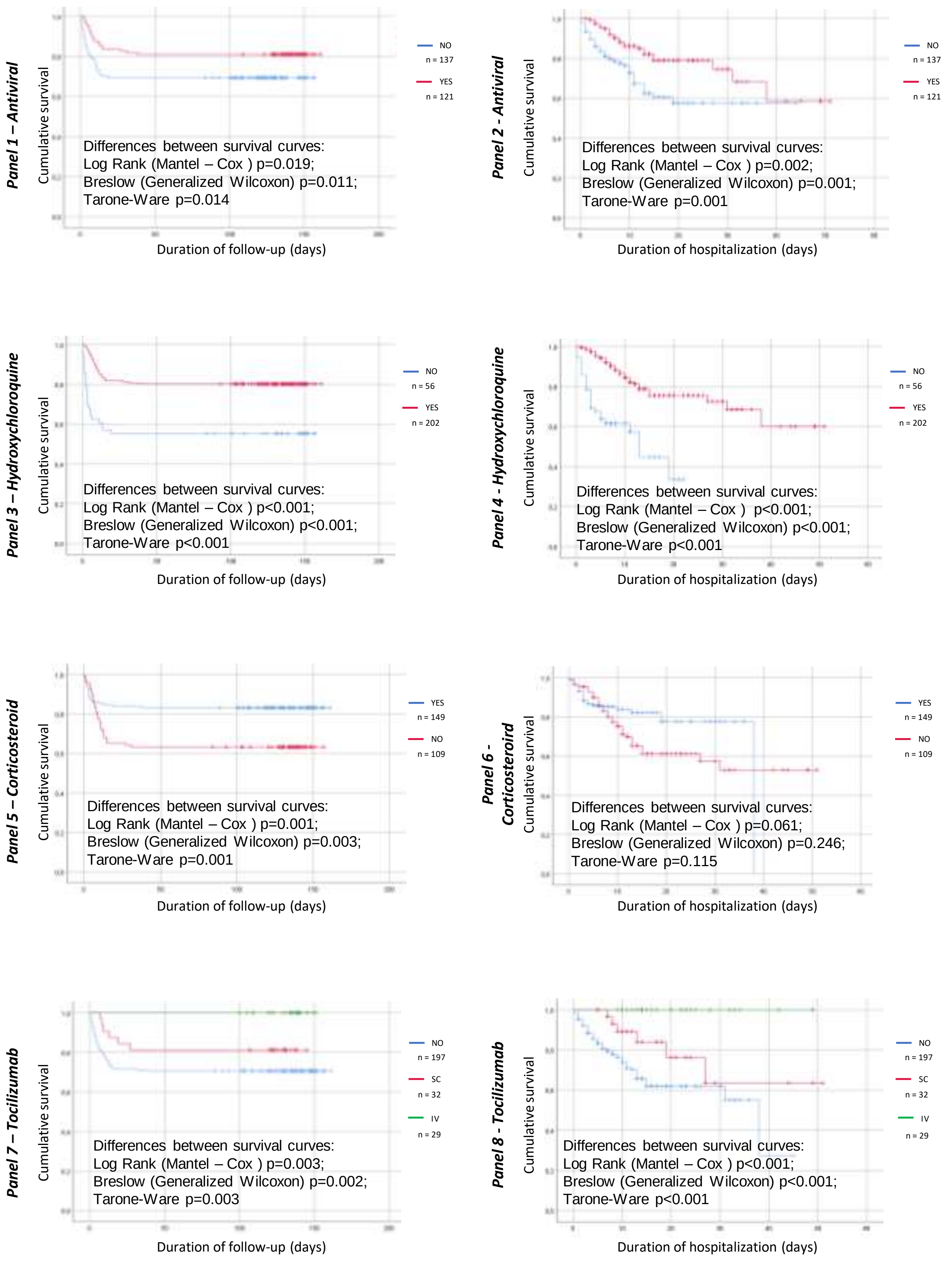

Figure legends

Figure 1 – Supplementary material
Legend as in Figure 4 of the main text

Figure 2 – Supplementary material
Kaplan-Meier survival curve for the different drug treatments during admission (excluding anticoagulant drugs)
Analysis run using group as factor; death as event and time to death/discharge or time to death/re-evaluation at follow up as time variable
Panel 1: treatment with lopinavir/ritonavir (red line) or no treatment with lopinavir/ritonavir (blue line), time to death/re-evaluation at follow up as time variable
Panel 2: treatment with lopinavir/ritonavir (red line) or no treatment with lopinavir/ritonavir (blue line), time to death/discharge as time variable
Panel 3: treatment with hydroxychloroquine (red line) or no treatment with hydroxychloroquine (blue line), time to death/re-evaluation at follow up as time variable
Panel 4: treatment with hydroxychloroquine (red line) or no treatment with hydroxychloroquine (blue line), time to death/discharge as time variable
Panel 5: treatment with dexamethasone (red line) or no treatment with dexamethasone (blue line), time to death/re-evaluation at follow up as time variable
Panel 6: treatment with dexamethasone (red line) or no treatment with dexamethasone (blue line), time to death/discharge as time variable
Panel 7: treatment with subcutaneous tocilizumab (red line), intravenous tocilizumab (green line) or no treatment with tocilizumab (blue line), time to death/re-evaluation at follow up as time variable
Panel 8: treatment with subcutaneous tocilizumab (red line), intravenous tocilizumab (green line) or no treatment with tocilizumab (blue line), time to death/discharge as time variable .
